# CAR-T-Cell Therapy for Solid Tumors Positive for Fibronectin Extra Domain B

**DOI:** 10.3390/cells11182863

**Published:** 2022-09-14

**Authors:** Zhijie Zhang, Chang Liu, Zhe Yang, Hongping Yin

**Affiliations:** School of Life Science and Technology, China Pharmaceutical University, Nanjing 211198, China

**Keywords:** fibronectin extra domain B, solid tumors, CAR-T-cells

## Abstract

(1) Background: The lack of specific targets has slowed the progress of CAR-T in treating solid tumors. Recent studies have revealed that EDB-FN (fibronectin extra domain B) may be an effective target for CAR-T treatment of solid tumors; EDB-FN is expressed in tumor and embryonic tissues, and antibody–cytokine fusion proteins targeting EDB-FN have been developed. However, the therapeutic effects of BBz CAR-engineered T-cells targeting EDB-FN in solid tumors have not been evaluated. (2) Results: In this study, we constructed a BBz CAR construct targeting EDB-FN, and the CAR molecule was expressed on the surface of T-cells by lentiviral transduction. In vitro, CAR-T-cells can be activated to express perforin and granzyme and lyse EDB-positive cells (U-87 MG cells, A549 cells, and HUVECs) and have no toxicity to EDB-negative cells (MCF-7). Compared to T-cells, CAR-T-cells can release cytokines after coculture with EDB-positive cell lines. In vivo, CAR-T-cells inhibited the progression of U-87 MG subcutaneous tumors and significantly reduced the blood vessel density in tumor tissue compared to T-cells, without obvious toxicity to mouse tissues and organs. Furthermore, CAR-T-cells overexpressing BiTE targeting EDB-FN can significantly improve their antitumor activity in vitro. (3) Conclusions: These results demonstrate that CAR-T-cells have specific antitumor and angiogenic activities in vivo and in vitro, suggesting that EDB-FN may be a potential solid tumor target for CAR-T therapy.

## 1. Introduction

Chimeric antigen receptor T-cells (CAR-T) are genetically engineered immune cells that are designed to express chimeric antigen receptors on their surface to recognize target-specific tumor-associated antigens and lead to tumor cell death. CAR-T therapy has been recognized as an effective method for treating tumors [1]. CAR-T-cells have been shown to have a good therapeutic effect in treating hematological malignancies, such as leukemia and lymphoma, leading to five approved CAR-T-cell therapies. Nonetheless, while solid tumor patients account for more than 90% of cancer patients [2], CAR-T therapy against solid tumors has been less effective than CAR-T therapy against hematological malignancies, with poor specificity for targets and poor coverage.

Compared with the success of CAR-T therapy in hematological malignancies, the development of CAR-T therapy in solid tumors has been slower. The unique challenges of solid tumors are characterized by tumor histopathology, lack of tumor-specific antigens, an immunosuppressive tumor microenvironment (TME), and life-threatening targeted extratumoral toxicity [3]. The lack of antigen specificity for target tumor cells is a key issue, leading to on-target and off-tumor toxicity in solid tumors [4]. Tumor-associated antigens (TAAs), i.e., antigens overexpressed on the surface of tumor cells, were initially considered to be an excellent target for CAR-T therapy, but their application resulted in the damage of normal healthy tissue throughout the body where these antigens are also present [5].

CAR molecules target tumor cell surface antigens. At present, the targets of cell therapy against solid tumors in clinical trials are mesothelin (MSLN) [6], mucin-1 (MUC1), human epidermal growth factor receptor 2 (HER2) [7], carcinoembryonic antigen (CEA) [8], tight junction protein 18.2 (claudin18.2), human epidermal growth factor receptor-2 (HER2), epidermal growth factor receptor variant III (EGFRvIII) [9], and so on. These clinical trials for the treatment of solid tumors have not achieved perfect treatment results, leading to safety risks even when tumor-associated antigens (TAAs) are expressed in small amounts in normal tissues. This limits the dose administered. Compared with treating hematological tumors, in solid tumors, CAR-T-cells first must infiltrate the tumor tissue, and the current proliferation activity of CAR-T-cells is not sufficient, implying that a larger dose is needed. However, the safety risks of targeting TAAs on the cell surface may lead to conservative doses in clinical treatment.

EDB-FN is a splice variant of extracellular fibronectin expressed in tumor tissues and embryonic tissues but not in normal tissues [10,11]. EDB-FN is expressed in a variety of solid tumors and lymphomas [12,13], e.g., liver cancer, breast cancer, lung cancer, colorectal cancer, melanoma, Hodgkin and non-Hodgkin lymphoma, glioma, and head and neck cancer [14]. Additionally, EDB-FN is not only expressed in tumor cells but also in tumor-associated fibroblasts and neovascular endothelial cells in the tumor microenvironment [15,16]. EDB-FN is related to the stability of tumor tissue structure and tumor angiogenesis [17] and has a positive correlation with the progression and malignancy of various tumors [18]. Therefore, EDB-FN, a secreted tumor-specific antigen, has a low safety risk and is a potential CAR-T therapeutic target.

L19 is an antibody targeting EDB-FN that has been used in clinical studies for IL-2 [19], IL-12, and TNF-α conjugate therapy in solid tumors [20]. Meanwhile, Wagner devised 28z CAR-engineered T-cells that exhibited anti-EDB-positive tumor activity [21]. Therefore, we first designed a CAR molecule (BBz) targeting EDB-FN, evaluated its in vitro and in vivo activities, and found that anti-EDB third-generation CAR-T and CAR-T-cells coexpressing bispecific T-cell engaging antibody (BiTE) also had antitumor activity.

## 2. Materials and Methods

### 2.1. Cell Lines and Culture

The human colorectal cancer cell line Caco2 and human breast cancer cell lines Hs 578t, MCF-7, and MDA-MB-468 were obtained from the Cell Bank of the Chinese Academy of Sciences (Shanghai, China). Caco-2 cells were cultured in MEM with 20% FBS. Hs 578t cells were cultured in DMEM with 0.01 mg/mL insulin and 10% FBS. MCF-7 cells were cultured in MEM with 0.01 mg/mL insulin and 10% FBS. MDA-MB-468 cells were cultured in Leibovitz’s L-15 medium with 10% FBS. The human glioblastoma cell line U-87 MG, lung cancer cell Line A549, mouse teratoma cell line F9, and mouse colorectal cancer cell line CT26 were acquired from Procell Life Science Co., Ltd. (Wuhan, China) U87MG cells were cultured in MEM with 10% FBS. A549 cells were cultured in Ham’s F-12K medium with 10% FBS. F9 cells were cultured in DMEM with 10% FBS. CT26 cells were cultured in 1640 medium with 10% FBS. The HUVEC line was purchased from Promocell and cultured in endothelial cell growth medium (C-22110, Promocell, Heidelberg, Germany). 293F cells were a gift from Dr. Yang and were cultured in 293 expression medium (12338018, Thermo Fisher Scientific, Waltham, MA, USA). Each vial of cells was subjected to subculture for up to two weeks after recovery.

### 2.2. Lentivirus Production

Human embryonic kidney 293F cells were seeded in a 250 mL cell shake flask for 24 h before transfection. Lentivirus was packaged using the packaging and envelope plasmids pLP1, pLP2, and pVSV-G in 293F cells. Plasmids were transferred into 293F cells by 293 ExpiFectamine (A14525, Thermo Fisher Scientific). The viral supernatant containing culture medium was obtained 48 h after transfection.

### 2.3. Transduction and Culture of Primary T-Cells

T-cells were isolated from peripheral blood mononuclear cells (PBMCs) using a negative magnetic selection kit according to the manufacturer’s protocol (130-096-535, Miltenyi, Bergisch Gladbach, Germany). T-cells were stimulated with magnetic beads coupled with anti-human CD3 and CD28 antibodies (11131D, Thermo Fisher Scientific). T-cells were cultured in complete RPMI medium (RPMI supplemented with 10% heat-inactivated fetal bovine serum and 100 U/mL recombinant human IL-2). Twenty-four hours after activation, T-cells were transduced with lentivirus and centrifuged at 2000 rpm for 60 min at 4 °C. BiTE lentivirus and CAR lentivirus were mixed 1:1 and co-transduced into T-cells to prepare CAR-T-cells overexpressing BiTE protein. The transduction efficiency was determined by flow cytometry. During the period of T-cell expansion, the cell concentration was maintained at 0.5 to 1 million cells/mL. The cells were expanded for 12 days and used for in vitro or in vivo assays.

### 2.4. Flow Cytometric Analysis

To analyze the transduction efficiency of T-cells, 10^6^ cells were incubated with 8 μg/mL reconstituted biotin-labeled polyclonal goat anti-human-F(ab)2 antibodies (109-066-097, Jackson Immunoresearch, West Grove, PA, USA) in FACS buffer (PBS with 0.4% FBS) for 25 min at 4 ℃. The cells were washed with FACS buffer and incubated with 5.5 µL phycoerythrin (R-phycoerythrin Streptavidin, 016-110-084, Jackson Immunoresearch) in FACS buffer for 20 min on ice in the dark. The cells were washed three times with ice-cold FACS buffer and analyzed by an ACEA Novocyte Flow Cytometer. Datas were further analyzed with NovoExpress Software v1.5.0 (Agilent, Santa Clara, CA, USA).

The expression of EDB-FN in U87MG and MCF-7 cells was detected by flow cytometry. 10^6^ cells were added with L19-FC antibody (5 μg/mL) and incubated for 30 min. Washed twice with DPBS buffer. It was then incubated with 1 μL of goat anti-human IgG Fc (DyLight 488) (ab97003, Abcam, Cambridge, UK) for 30 min in the dark. Cell suspensions were washed with DPBS and detected by flow cytometry.

Mock and CAR-T-cells were stained with CellTrace™ Far Red (C34564, Thermo Fisher Scientific) according to the manufacturer’s instructions. T-cells were co-cultured with U87MG cells 1:1, and the fluorescence intensity of T-cells was detected on Day 0 and Day 5.

Mock and CAR-T-cells were co-cultured 5:1 with U87MG cells for 4 h. T-cells were stained with APC anti-human CD69 antibody (310910, Biolegend, San Diego, CA, USA) and detected by flow cytometry.

### 2.5. CD107a Degranulation Assay

Mock and CAR-T-cells were co-cultured with U87MG cells in effector-to-target ratios of 5 in a 96-well U-bottom plate. FITC anti-human CD107a (LAMP-1) antibody (328606, Biolegend) was added to the co-culture for 1 h. Then, according to the instructions, monensin was added and incubated for 3 h and detected by flow cytometry.

### 2.6. Quantitative Real-Time PCR

Total RNA was extracted from target cells (19221, Yeasen, Shanghai, China), and the transcriptional synthesis of cDNA was reversed using total RNA as a template (11121ES60, Yeasen). GADPH F-primer 5′-ACCCAGAAGACTGTGGATGG-3′ and R-primer 5′-TCTAGACGGCAGGTCAGGTC-3′; EDB F-primer: 5′-AACTCACTGACCTAAGCT TT-3′ and R-primer 5′-CGT TTG TTG TGT CAG TGT AG-3′. cDNA was used as a template using the SYBR Green dye method for qPCR (11199ES03, Yeasen).

### 2.7. Cytotoxicity and Cytokine Release Assays In Vitro

For cytotoxicity and cytokine induction of CAR-T-cells, the target cells were mixed with transduced T-cells in effector-to-target ratios of 10, 5, and 1 in a 96-well U-bottom plate. After 24 h of culture, target cell lysis was detected by an LDH detection kit (40209ES76, Yeasen). Interferon-gamma expression was measured using ELISA (110002, DAKEWE, Beijing, China). Mock and CAR-T-cells were co-cultured 5:1 with U87MG cells for 24 h. Granzyme B expression was measured using ELISA (DKW12-1850, DAKEWE).

### 2.8. Animal Models

Six- to eight-week-old female NOD/ShiLtJGpt-Prkdcem26Cd52Il2rgem26Cd22/Gpt (NCG) mice were obtained from GemPharmatech Co., Ltd. (Nanjing, China) and housed in SPF conditions (China Pharmaceutical University). All animal experimental protocols were reviewed and approved by the Institutional Animal Care and Use Committee of the Center for New Drug Safety Evaluation and Research, China Pharmaceutical University (Approval Code: 2022-03-28-1). In the U-87 MG subcutaneous tumor model, ten NCG mice were subcutaneously implanted with 10^6^ U-87 MG cells, and, at 14 days, they were divided into two groups according to tumor volume. A total of 5 × 10^6^ T-cells or CAR-T-cells were injected into the tail vein on Days 15, 22, and 29. In addition, tumor sizes were monitored every 2–3 days (V = (L × W × W)/2). On Day 38, the mice were sacrificed, and the tumors were extracted and weighed.

For the in vivo toxicity evaluation experiment, 12 eight-week-old NCG mice were randomly divided into three groups: T-cell group (*n* = 5); EDB-CAR-T-cell group (*n* = 5); and high-dose group (*n* = 2). One dose of 1 × 10^7^ T-cells or 1 × 10^7^ or 2 × 10^7^ EDB CAR-T-cells was intravenously injected into the tail vein in 200 μL of physiological saline. The body weight and survival status of the mice were monitored once every two days. All mice were sacrificed on Day 21 following T-cell infusion to harvest various tissues, which were then formalin-fixed, paraffin-embedded, and stained with hematoxylin–eosin.

### 2.9. Immunohistochemistry (IHC) Analysis

Briefly, tissue sections were deparaffinized and blocked with goat serum before incubation overnight at 4 °C with CD31 or BC−1 antibodies. After washing, the cells were incubated with horseradish peroxidase-conjugated secondary antibody for 2 h at room temperature. The cells were then incubated with a chromogenic substrate for 10 min and visualized under a microscope.

### 2.10. Statistical Analysis

All data are presented as the mean ± SD. An unpaired two-tailed Student’s *t*-test was used to determine the statistical significance of the two-sample comparisons of in vitro and in vivo experiments. All statistical analyses and graph plottings were carried out utilizing GraphPad Prism 8.0 software (GraphPad Software, La Jolla, CA, USA).

## 3. Results

### 3.1. EDB-FN Antigen Is Expressed in Various Tumors and Related Cells

**Figure 1:**
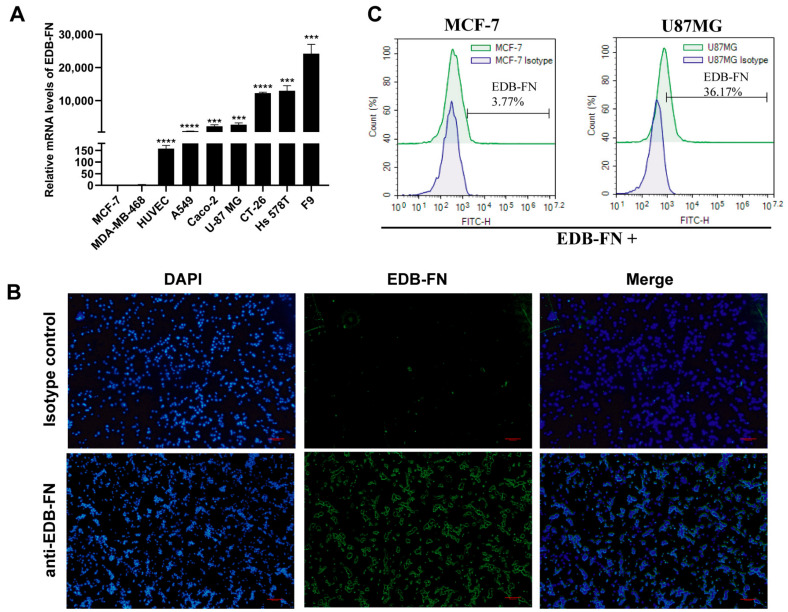
Analysis of EDB-FN expression in human cancer cells and HUVECs. (**A**) EDB-FN protein expression levels in different cells detected by qPCR. (**B**) The EDB-FN expression test on U-87 MG tumor cells by immunofluorescence staining. (**C**) EDB-FN expression test on U-87 MG and MCF-7 tumor cells by flow cytometry. Datapoints reflect the mean ± SD of triplicates (*** *p* < 0.001; **** *p* < 0.0001; two-tailed Student’s *t*-test).

EDB-FN protein is a splice variant of FN protein expressed in embryonic and tumor tissue. EDB-FN expression was detected in cancer cells, such as U-87 MG, A549, Caco-2, and Hs 578t cells, but not in MCF-7 cells by qPCR. Intriguingly, the EDB-FN expression level in HUVECs was consistent with that in the corresponding A549 cells (Figure 1A). EDB-FN protein is secreted by cells into the intercellular space and is an important component of the extracellular matrix. EDB-FN protein can bind to integrins on the cell membrane surface through the arginine-glycine-aspartic acid (RGD) motif [22]. By immunofluorescence analysis, we found that EDB-FN was present around U87MG cells (Figure 1B). Furthermore, we detected EDB-FN protein on the surface of U-87 MG cells by flow cytometry, but no signal in MCF-7 cells (Figure 1C). These results suggest that EDB-FN may be secreted in the intercellular substance but bound to the cell membrane surface. 

### 3.2. CAR Construction and Characteristics

**Figure 2:**
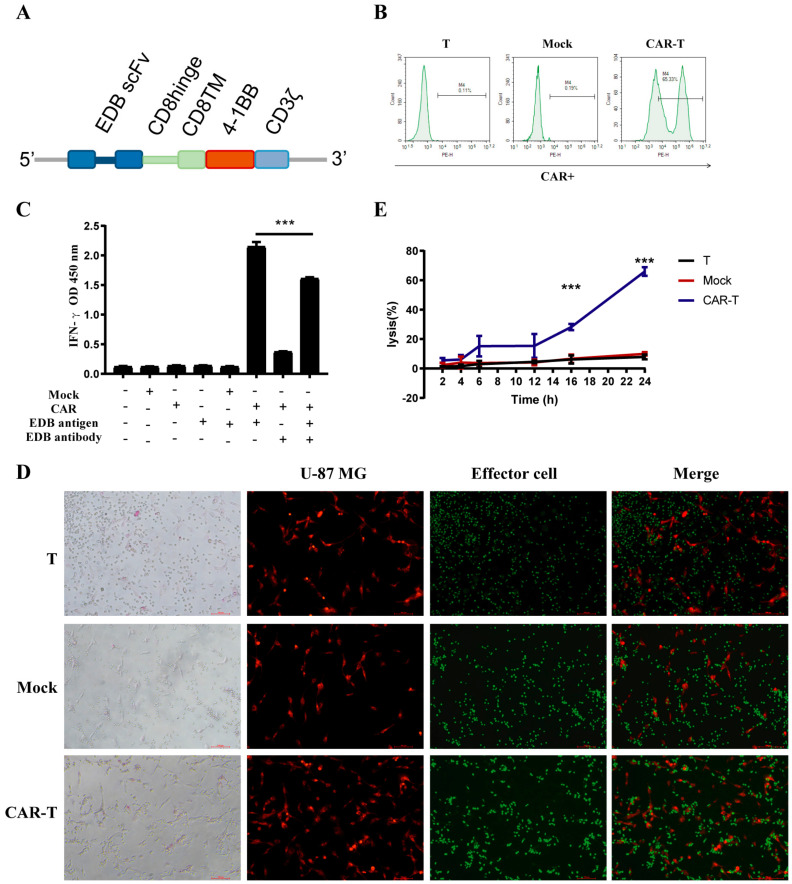
Characterization of EDB-FN-targeted CAR-T-cells. (**A**) Schematic representation of EDB-FN-targeted CAR. The L19-BBz28Z CAR comprises an scFv, human CD8a hinge, and transmembrane (TM) region and the intracellular signaling domains from human 4−1BB and CD3z. (**B**) Expression of EDB-FN-targeted CAR and mock transduction in lentivirus-transduced human T-cells was analyzed using flow cytometry. The transduction efficiency is shown. (**C**) T-cells were transduced with the EDB-FN-targeted CAR lentivirus. Cells were then incubated with recombinant EDB-FN protein and EDB-FN antibody to test EDB-FN protein-activating EDB-FN CAR-T-cells by detecting IFN-γ in the culture by ELISA. (**D**) EDB-FN CAR-T (CFSE) cells cocultured with U-87 MG (Dil) cells (Effector cells: Target cells = 5) for 4 h. The cells were observed with a fluorescence microscope. (**E**) EDB-FN CAR-T-cells cocultured with U-87 MG cells at (E:T) 5 for 2–24 h. Cell lysis was determined using an LDH assay. Data are representative of three independent experiments. Datapoints reflect the mean ± SD of triplicates (*** *p* < 0.001; two-tailed Student’s *t*-test).

We first generated an EDB-targeting CAR molecule containing 4-1BB and CD3z domains. Anti-EDB-FN CAR (BBz) constructs comprised L19 scFv linked to a CD8α hinge and transmembrane region, followed by a 4-1BB intracellular signaling motif and in tandem with the CD3ζ signaling moiety (Figure 2A). Primary T-cells were transduced with lentivirus encoding the CAR genes to generate CAR-T-cells. Mock T-cells were also made by transfection with empty vector lentivirus. The transduction efficiency of mock and CAR-T-cells was 0.19% and 65.33%, respectively (Figure 2B).

### 3.3. CAR-T-Cells Exert Antigen-Specific Cytotoxic Potency and Cytokine Secretion In Vitro

**Figure 3:**
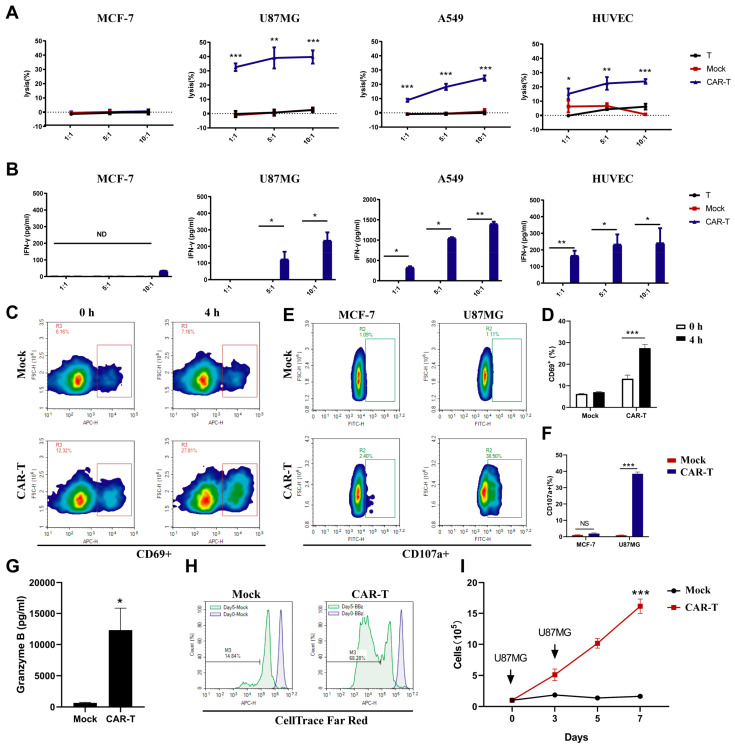
Evaluation of EDB-FN-targeted CAR-T-cell reactivity to cancer cells. (**A**) In vitro cytotoxic activities of EDB-targeted CAR-T-cells. Primary human T-cells transduced with the indicated lentiviral vectors were incubated with the cells with different EDB-FN expression levels at various E:T ratios for 24 h. Cell lysis was determined using an LDH assay. (**B**) In vitro IFN-γ production by EDB-targeted CAR-T-cells in the presence of tumor cells. EDB CAR-T-cells were incubated with cancer cells with different EDB-FN expression levels at various E:T ratios for 24 h. Culture supernatants harvested at 24 h after coculture were assayed for IFN-γ. (**C**,**D**) The antigen-specific activation of EDB-targeted CAR-T-cells. U87MG cells were co-cultured with mock or CAR-T-cells for 4 h. The CD69 marker was detected by flow cytometry. (**E**–**G**) The expression of perforin and granzyme of EDB-targeted CAR-T-cells induced by antigen. U87MG cells were co-cultured with mock or CAR-T-cells for 24 h. The CD107a marker detected by flow cytometry and granzyme B detected by ELISA. (**H**,**I**) The proliferation of EDB-targeted CAR-T-cells. (**H**) Mock and CAR-T-cells stained with CellTrace and U87MG cells were co-cultured, and the fluorescence intensity of cells was detected by flow cytometry on Days 0 and 5. (**I**) T-cell proliferation under repeated antigen stimulation; arrows represent stimulation using U87MG cells on Days 0, 3, 5, and 7. (**F**) Cell numbers were counted on Days 0, 2, 5, and 7. Data are representative of three independent experiments. Datapoints reflect the mean ± SD of triplicates (* *p* < 0.05; ** *p* < 0.01; *** *p* < 0.001; two-tailed Student’s *t*-test).

Next, we examined the function of anti-EDB-FN CAR in primary T-cells, and T or mock cells served as the negative control. The killing of U-87 MG cells by anti-EDB-FN CAR-T-cells was also time-dependent, and the cleavage rate of U-87 MG cells exceeded 60% at 24 h (Figure 2C). Compared to the T-cell and mock groups, CAR-T (BBz) cells were specifically activated by EDB antigen to express IFN-γ, and the level of IFN was inhibited by L19 antibody addition (Figure 2D). Effector cells were labeled with green fluorescence, and U-87 MG cells were labeled with red fluorescence. After coculturing for 4 h with an effector-to-target ratio of 5:1, the CAR-T-cells, but not T or mock cells, were closely surrounded by U-87 MG cells (Figure 2E). These data indicated that anti-EDB-FN CAR-T-cells could be activated by EDB-FN antigen. The anti-EDB-FN CAR-T-cells efficiently lysed EDB-positive U-87 MG cells, A549 cells, and HUVECs but not EDB-negative MCF-7 cells (Figure 3A). T and mock T-cells showed very weak cytotoxicity compared to anti-EDB-FN CAR-T-cells. Similarly, IFN-γ release after 24 h of coculture revealed that anti-EDB-FN CAR-T-cells produced more cytokines than the negative control (Figure 3B). After stimulation with U87MG cells, the activation of CAR-T-cells was more significant than that of T-cells by detecting the up-regulation of CD69 (Figure 3C,D). Because the expression of perforin and granzyme is the main way for T-cells to exert their cytotoxic function, we also evaluated the expression of CD107a and Granzyme B in T- and CAR-T-cells. Co-cultured with U87MG, CAR-T-cells expressed more CD107a and Granzyme B than T-cells (Figure 3E–G). While co-cultured with EDB-FN negative cells (MCF-7), there was no difference in the proportion of CD107a positive cells between CAR-T-cells and T-cells (Figure 3E,F). We next examined antigen-dependent proliferation of CAR-T-cells. Far Red-stained CAR-T-cells and cell number counting reflect the ability of CAR-T-cells to proliferate after antigen stimulation (Figure 3H,I). These data indicated that these CAR-T-cells had intrinsic target-dependent cytotoxic activity and proliferation.

### 3.4. CAR-T-Cells Inhibit U-87 MG Tumor Progression In Vivo

**Figure 4:**
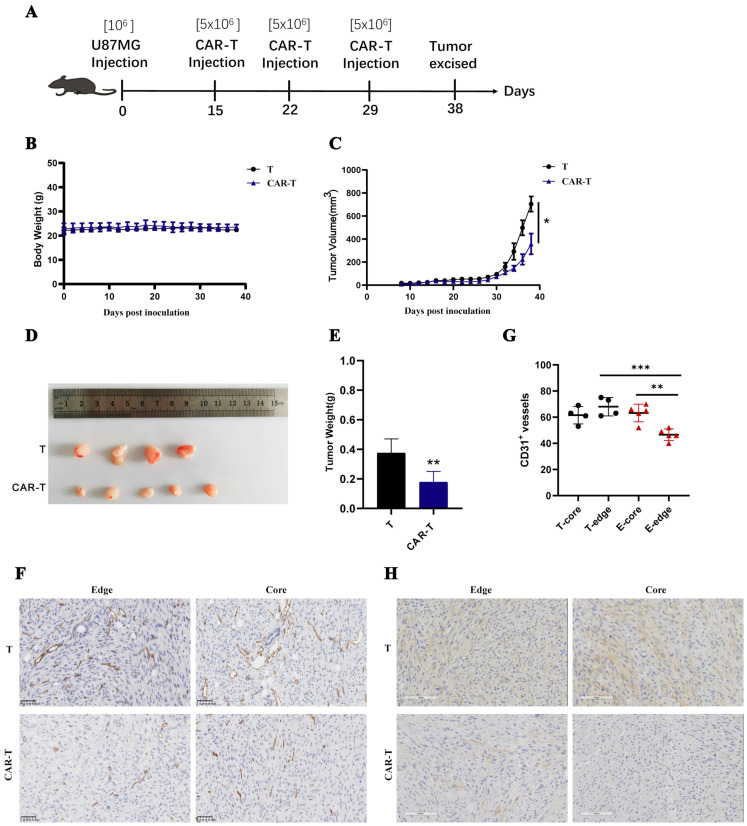
In vivo antitumor activities of EDB-FN-targeted CAR-T-cells on established subcutaneous glioblastoma xenografts. (**A**–**C**) NCG mice were subcutaneously inoculated with 10^6^ U-87MG cells on Day 0. Three doses of 5 × 10^6^ EDB-FN-targeted CAR-T-cells or T-cells were intravenously administered on Days 15, 22, and 29. Data are presented as the mouse mean weight and mean tumor volume and SEM. (**D**,**E**) On Day 38 after tumor cell inoculation, the mice were euthanized. Tumor weight was measured. (**F**,**G**) The expression of mouse vessels (CD31 protein) was tested in the tumor tissue using IHC assays. (**H**) The expression of EDB-FN protein was tested in the tumor tissue using IHC assays. Datapoints reflect the mean ± SD of triplicates (* *p* < 0.05; ** *p* < 0.01; *** *p* < 0.001; two-tailed Student’s *t*-test).

A solid tumor model was used to test the in vivo antitumor efficacy of anti-EDB-FN CAR-T-cells. In the EDB+ U-87 MG epithelial morphology carcinoma model, 10^6^ U-87 MG cells were subcutaneously inoculated into nonobese diabetic/ShiLtJGpt-Prkdcem26/Il2rgem26/Gpt (NCG) mice, and T-cells were intravenously infused on Days 15, 22, and 29 after tumor inoculation (Figure 4A). During the treatment period, the weight of mice in the CAR-T-cell group was not different from that in the T-cell group, indicating that CAR-T-cells did not cause severe toxicity in the mice (Figure 4B). On Day 38, the tumor volume and weight of mice in the CAR-T-cell group were significantly lower than those in the T-cell group, indicating that CAR-T-cells had an inhibitory effect on the progression of U-87 MG subcutaneous tumors (Figure 4C–E). Since CAR-T-cells had a killing effect on HUVECs in vitro, the number of blood vessels in mouse tumor tissue was used to characterize blood vessel destruction in tumor tissue by CAR-T-cells. The immunohistochemical analysis of CD31 in tumor tissue sections showed that CAR-T-cells significantly reduced the blood vessel density in the center and edge of the tumor tissue. The blood vessels at the edge of the tumor tissue were particularly destroyed (Figure 4F,G). The failure of CAR-T-cells to regress tumor tissue may be due to reduced EDB-FN antigen in tumor tissue. Immunohistochemical staining revealed that the amount of EDB in the interior and edge of the tumor tissue in the CAR-T-cell group was less than that in the T-cell group (Figure 4H). These results suggest that anti-EDB-FN CAR-T-cells inhibited EDB-positive glioma cancer xenografts in vivo.

### 3.5. On-Target Off-Tumor Toxicities in Mouse Models Treated with CAR-T-Cells

**Figure 5:**
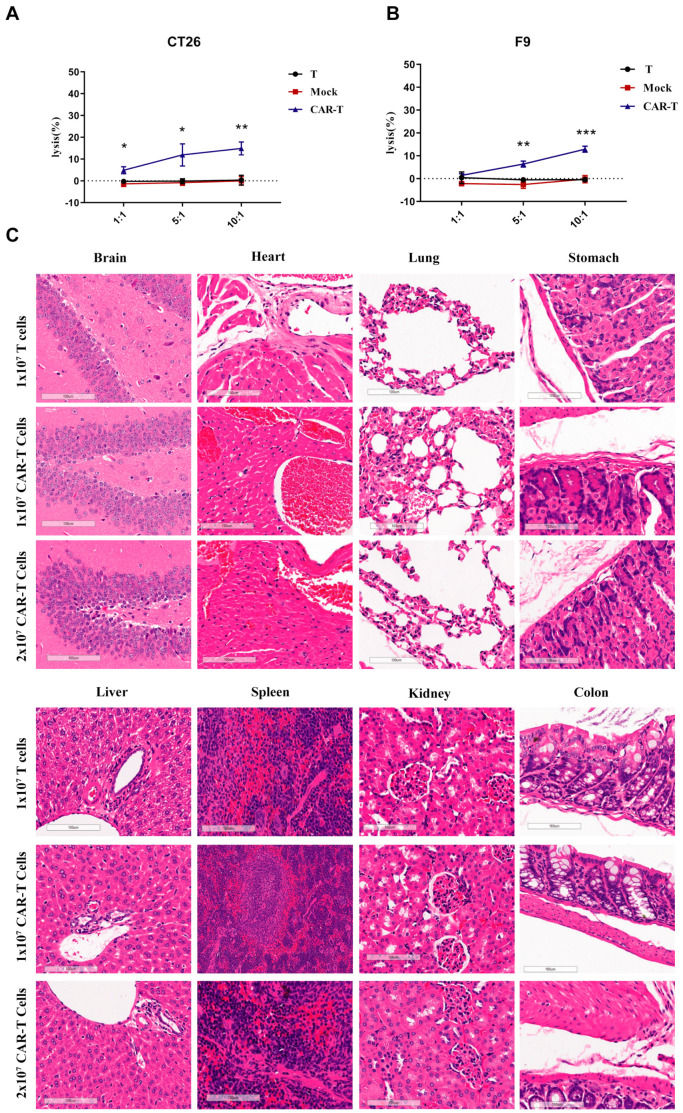
Histopathological analysis of murine organ tissues by hematoxylin and eosin staining. (**A**,**B**) Human EDB-targeted CAR-T-cells incubated with mouse tumor F9 or CT26 cells for 24 h. Cell lysis was determined using an LDH assay. (**C**) NCG mice were treated with 1 × 10^7^ T-cells and 1 × 10^7^ or 2 × 10^7^ EDB CAR-T-cells and sacrificed on Day 21 following T-cell infusion. Different tissues were harvested, formalin-fixed, paraffin-embedded, and stained with H&E. Representative photomicrographs are shown. The images were taken through a Leica Aperio VERSA 8 slice scanner under 20× magnification. Each scale bar represents 100 μm. No obvious pathological changes were found in any tissues, and there were no significant differences between all groups. Datapoints reflect the mean ± SD of triplicates (* *p* < 0.05; ** *p* < 0.01; *** *p* < 0.001; two-tailed Student’s *t*-test).

Since the human EDB-FN protein is the same as the mouse EDB-FN protein and anti-EDB CAR-T-cells can be activated by free EDB antigen, it is necessary to clarify the toxicity of anti-EDB CAR-T-cells in normal mouse tissues and organs. Both mouse teratoma cells (F9) and mouse colorectal cancer cells (CT26) express EDB protein; therefore, we first examined the cytotoxic activity of CAR-T-cells against F9 cells and CT26 cells. As displayed in Figure 5A,B, CAR-T-cells had cytotoxic effects on F9 cells and CT26 cells when the effector-to-target ratio was 5:1 and 10:1, while the T-cell group and mock cell group showed almost no cytotoxic effects. NCG mice were injected with 10^7^ T-cells or 10^7^ or 2 × 10^7^ CAR-T-cells through the tail vein. Surprisingly, no obvious tissue damage was observed in the mouse heart, lung, or other tissues and organs (Figure 5C). Taken together, these data support that anti-EDB CAR-T-cells did not induce on-target off-tumor toxicities in the in vivo mouse models.

### 3.6. Enhanced Signaling Improves CAR-T-Cell Cytotoxicity against U87MG Cells

**Figure 6:**
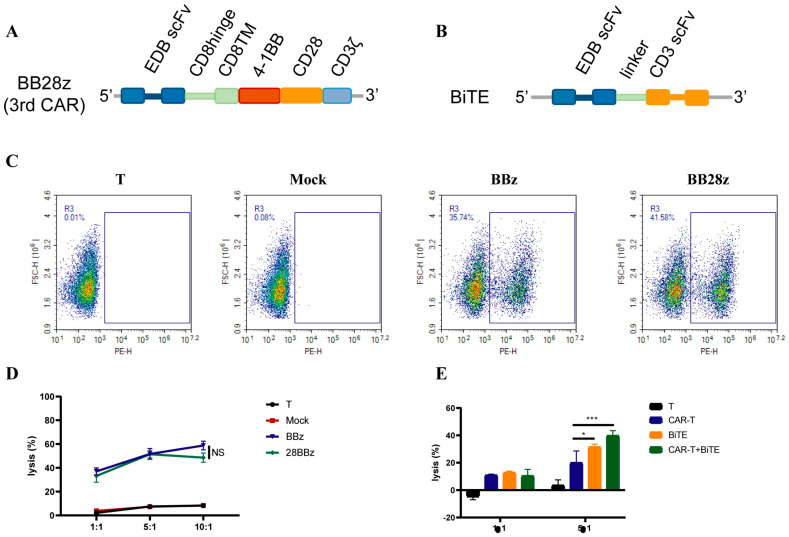
Enhanced cytotoxicity of CAR-T-cells against U87MG cells. (**A**,**B**) Schematic representation of EDB-FN-targeted CARs. The BB28z (3rd) CAR comprises an scFv, human CD8a hinge, and transmembrane (TM) region, and the intracellular signaling domains from human 4−1BB, CD28, and CD3z. BiTE comprises L19 scFv and OKT3 scFv in tandem via a linker. (**C**) The transduction efficiency of BBz (2nd) and BB28z (3rd) is shown. (**D**) In vitro cytotoxic activities of 3rd CAR-T-cells. (**E**) In vitro cytotoxic activities of BiTE-expressing T and CAR-T-cells. Data are representative of three independent experiments. Datapoints reflect the mean ± SD of triplicates (* *p* < 0.05; *** *p* < 0.001; two-tailed Student’s *t*-test).

To enhance the killing activity of CAR-T-cells, we constructed a third-generation CAR molecule by concatenating CD28 on the BBz CAR molecule (Figure 6A). However, there was no significant difference in the toxicity of U-87 MG cells between the second and third generations of CAR (Figure 6B). Considering that a CD3e antibody (OKT3) can activate T-cells, a BiTE antibody targeting EDB in CAR-T-cells was overexpressed. CAR-T-cells overexpressing BiTE increased the killing of U-87 MG cells by approximately 50% in vitro, suggesting that TCR signaling pathway activation can significantly enhance the cytotoxic activity of CAR-T-cells (Figure 6C).

## 4. Discussion

CAR-T-cell immunotherapy has been successfully applied to treat hematological tumors, with five CAR-T-cell products commercially available to treat B-cell lineage hematological malignancies, including Novartis’ Kymriah, Gilead’s Yescarta and Tecartus, and Bristol-Myers Squibb’s Breyanzi and Abecma, which were jointly developed by Bristol-Myers Squibb and Bluebird Bio. Despite the slow progress of CAR-T in solid tumor therapy, a CAR-T clinical trial targeting a novel antigen, claudin18.2, showed that the objective response rate and disease control rate of 37 patients with advanced gastrointestinal tumors reached 48.6% and 73.0%, respectively [23]. Therefore, developing new targets is important for treating solid tumors with CAR-T-cells.

EDB-FN protein is expressed in tumor and embryonic tissues. In clinical studies, cytokines or radioactive elements were conjugated with EDB-FN-targeting antibodies to deliver drugs to tumor sites for treatment [20]. Therefore, we envision using EDB-FN as a target for studying cellular immunotherapy. We confirmed that EDB-FN is expressed in human glioma cells, lung cancer cells, colorectal cancer cells, breast cancer cells, and HUVECs but is expressed at low levels in MCF-7 cells. Although EDB-FN is a secreted protein, it was found close to U87MG cells, indicating that CAR-T-cells could contact tumor cells to exert cytotoxic effects. The in vitro experiments showed that T-cells expressing CAR molecules with BBz structures could directly contact the target cells, and the killing of target cells depends on EDB expression. In vivo, CAR-T-cells significantly inhibited the progression of U87MG subcutaneous tumors and reduced the vascular density in tumor tissue. Immunohistochemical analysis revealed that EDB-FN content in the tumors of mice in the CAR-T treatment group decreased. Since human and mouse EDB-FN are the same, CAR-T-cells also have a killing effect on mouse tumor cells. Fortunately, no obvious damage was observed in the tissues and organs of mice administered 20 million EDB-FN CAR-T-cells, indicating that EDB-FN expression has specific and cytotoxic effects; therefore, administration of EDB-FN CAR-T-cells is a highly specific and safe treatment.

CD28 or 4-1BB molecule is commonly used as a second signal in second-generation CAR structures. In the design of CAR molecules targeting EDB-FN, Wagner created the 28z CAR [21], and we designed the BBz CAR. These structures targeting EDB-FN differ in cellular activity. For example, compared to 28z CAR-T-cells, BBz CAR-T-cells secreted fewer IFN-γ cytokines after activation. Less cytokine expression may increase the safety of CAR-T-cell therapy in vivo but may also reduce efficacy. Despite the structural differences, both strategies inhibited EDB-FN-positive tumors.

Our findings suggest that EDB-FN-targeting BBz CAR-T-cells have specific cytotoxic effects on EDB-FN-positive cell lines. In treating subcutaneous U87MG tumors in mice, tumor progression is inhibited by killing target cells and tumor blood vessels, but there was no EDB-FN-control in our in vitro cytotoxicity experiments (MCF-7 cells also have a small amount of EDB-FN expression). Simultaneously, EDB-FN did not cause tumor regression in the in vivo antitumor and in vitro cytotoxicity experiments. Even though the effector-to-target ratio reached 10, it could incompletely eliminate tumor cells. This might be because EDB-FN is a secreted protein, despite being present around the cell, which is distinct from a membrane protein. For example, when CAR-T-cells target membrane proteins, another receptor—ligand binding occurs between effector cells and target cells to enhance CAR-T-cell activation. Therefore, we attempted to improve the activity of EDB-FN CAR-T-cells by enhancing the activation signal of CAR-T-cells. Although the 3-generation CAR by tandem second signal failed to achieve its purpose, CAR-T-cells enhanced the antitumor activity of CAR-T-cells through the TCR-CD3 complex by coupled expression of BiTE.

## 5. Conclusions

In conclusion, EDB-FN may serve as a general target for treating solid tumors, and, based on our results, solid tumors can be treated with EDB-FN CAR-T-cells.

## Data Availability

Not applicable.
